# Adverse Maternal and Perinatal Outcomes of Meconium-Stained Amniotic Fluid in Term Labor at Hospitals in South Gondar Zone, Northwest Ethiopia: A Prospective Cohort Study

**DOI:** 10.1155/2023/8725161

**Published:** 2023-08-04

**Authors:** Dagne Addisu, Maru Mekie

**Affiliations:** Department of Midwifery, College of Health Sciences, Debre Tabor University, Ethiopia

## Abstract

**Background:**

The passage of meconium during labor increased the chance of undesirable birth outcomes. The adverse effects of meconium are worsening in resource-limited countries. In Ethiopia, there is an argument concerning meconium's negative effects and management on pregnant women and their babies. Therefore, this study was intended to assess the adverse maternal and perinatal outcomes of meconium in term labor in the South Gondar Zone, Ethiopia.

**Methods:**

A prospective cohort study was conducted using 580 laboring mothers (145 exposed and 435 nonexposed groups). A two-stage sampling method was implemented to get study subjects. The data were collected using an interviewer-administered structured questionnaire and a medical chart review. SPSS version 25 was used for data analysis. Chi-squared and Fisher's exact tests were used to compare the two groups' differences. The strength of the association was measured using relative risk with a 95% CI.

**Result:**

There was more operative delivery (28.3% versus 5.3%), puerperal sepsis (79.54% versus 2.06%), nonreassuring fetal heart rate pattern (29.7% versus 2.1%), meconium aspiration syndrome (7.58% versus 0.68%), neonatal sepsis (9% versus 4.1%), perinatal asphyxia (13.8% versus 7.6%), admission to the neonatal intensive care unit (23.4% versus 3.2%), and early neonatal deaths (4.8% versus 1.4%) among meconium stained groups as compared to the clear amniotic fluid groups.

**Conclusion:**

Meconium-stained amniotic fluid significantly increased adverse maternal and perinatal outcomes in Ethiopia. The risk of perinatal asphyxia, nonreassuring fetal heart rate pattern, neonatal sepsis, meconium aspiration syndrome, admission to the NICU, early neonatal death, operative delivery, and puerperal sepsis were significantly higher in meconium-exposed groups. Special attention should be given to meconium-exposed mothers during the intrapartum period and in postnatal follow-up.

## 1. Introduction

Meconium is a newborn's initial stool or excrement, which comprises desquamated cells, intestinal mucus, vernix caseosa, adipose tissue, and digestive excretions [1, 2]. Meconium begins to form in the fetal gut around week 12 of conception and then deposits in the fetal bowel for the duration of the pregnancy [3, 4]. It is frequently detected in the labor ward due to different reasons, and its occurrence may confuse health professionals. It could occur physiologically as a result of gastrointestinal tract maturation or pathologically as a result of acute or chronic hypoxia [5, 6].

Meconium usually passes over the first 24 to 48 hours following delivery. Meconium, however, can pass during labor for a variety of reasons. Some of the factors responsible for the passage of meconium were obstructed labor, advanced maternal age, tobacco smoking, oligohydramnios, anemia, prolonged duration of labor, use of a uterotonic agent during labor, and hypertensive disorders of pregnancy [2, 7, 8].

The prevalence of intrapartum MSAF in term pregnancy varies across regions and ranges from 7% to 22%. The rate could increase to 40% in postterm pregnancies [9, 10]. In Ethiopia, the prevalence varied from 15.47% to 17.8% [2, 11].

MSAF significantly increases the chance of undesirable birth outcomes. Some of these outcomes were intrapartum fetal death, neonatal sepsis, meconium aspiration syndrome (MAS), and early neonatal death (END) [10, 12–14]. In the world, between 5 and 10.5% of newborns with MSAF acquire MAS, which has a 13% death rate and a 40% case fatality rate [15]. Furthermore, children born with MSAF had greater rates of serious mental impairment and intellectual disabilities [15, 16]. Moreover, MSAF also leads to serious maternal morbidities such as intrapartum chorioamnionitis and postpartum endometritis [11, 12]. Adverse outcomes are worsening in resource-limited countries due to various structural factors and healthcare quality [17].

Adverse maternal and perinatal outcomes can be reduced through closely monitoring intrapartum and postpartum conditions and by providing evidence-based prevention and management for meconium-associated complications [7, 18]. However, there are some unresolved arguments concerning the outcome and management of MSAF in Ethiopia (different concerns regarding the mode of delivery, the need for fluid resuscitation for the mother in the case of MSAF, and the need for neonatal evaluation after delivery).

Even though various studies have described the negative effects of MSAF on pregnant women and their babies in developed countries, there is limited evidence in Ethiopia. Thus, this research was intended to assess the adverse maternal and perinatal outcomes of MSAF in term labor in the South Gondar Zone, Northwest Ethiopia. This research showed the negative effects of MSAF in Ethiopia and will help the Federal Minister of Health develop treatment protocols and preventive measures. In addition, obstetricians and midwives will be familiar with the effects of MSAF and will monitor high-risk mothers strictly during the intrapartum and postpartum periods. The findings of this study will also act as baseline data for future research.

## 2. Materials and Methods

This manuscript was prepared and reported in accordance with the STROBE Guideline for cohort studies.

### 2.1. Study Design and Setting

A prospective cohort study was carried out between April and August 2021 at hospitals in the South Gondar Zone, Ethiopia. This zone has 8 hospitals (1 comprehensive specialized hospital and 7 primary hospitals). Two additional primary hospitals were constructed in the zone and have been functional since February 2022.

### 2.2. Study Population and Eligibility Criteria

All mothers with MSAF during labor at selected hospitals in the South Gondar Zone during the study periods were the study population for the exposed group, while mothers with clear amniotic fluid during labor were the study population for the nonexposed group. The study included every pregnant woman with a singleton pregnancy, cephalic presentation, and ruptured fetal membranes. This study excluded mothers whose fetus died intrauterine before the onset of labor.

### 2.3. Sample Size Determination

Different outcomes from previous studies were considered to calculate the sample size. A total of 580 study participants were used in this study (145 MSAF cases and 435 CAF cases). The sample size was computed by Epi Info version 7.0 using the following hypotheses: 6.1% and 19.7% of the nonexposed and exposed groups, respectively (taken from the previous study), with a 95% CI, 80% of the power, and exposed to the nonexposed ratio of 1 : 3, 10% nonresponse rate, and a design effect of 2.

### 2.4. Sampling Strategies

A two-stage sampling procedure was implemented to get MSAF and CAF cases. Initially, four hospitals out of the eight were chosen using a random sample method (Adis Zemene, Debre Tabor, Mekane Eyesus, and Nefas Mewcha). By referring to the flow of cases, a number of study participants were proportionately distributed to each hospital. In the second stage, a systematic random sampling technique was applied to identify nonexposed groups. A sequential sampling strategy was implemented for the selection of MSAF cases. As a result, every MSAF case during labor was selected until we achieved a sufficient number.

### 2.5. Variable of the Study

The dependent variables were maternal and perinatal outcomes, whereas the independent variables include maternal sociodemographic and obstetric characteristics such as age, residence, woreda/kebele (smallest administrative unit), educational status, occupation, marital status, number of pregnancies, number of births, ages of gestation (GA), ANC visit, mode of delivery, the status of amniotic fluid, duration of membrane rupture, any obstetric or medical disease, grade of meconium, sex, and weight of the fetus.

### 2.6. Outcome Measures and Definition of Terms

The exposed groups were mothers who had meconium-staining fluid during labor, while the nonexposed groups were laboring mothers who had clear amniotic fluid throughout labor [17]. The presence of meconium was detected immediately after the rupture of the membrane. A mother who had initially clear amniotic fluid but developed meconium in the late stage of labor was considered to be in the exposed group.

Adverse Maternal Outcomes: Maternal outcomes during labor and within the first 7 days after delivery mainly chorioamnionitis, operative delivery, puerperal infections, and wound infection in the exposed (MSAF) groups were compared to the nonexposed (clear amniotic fluid) groups .

Adverse Perinatal Outcomes: Neonatal outcomes during labor and within the first 7 days after delivery mainly nonreassuring fetal heart rate pattern, the need for neonatal resuscitation, admission into the NICU, intrapartum stillbirth, MAS, perinatal asphyxia, END, and neonatal sepsis in the exposed (MSAF) group were compared to the nonexposed (CAF) groups .

Early Neonatal Death: Death of a neonate within the first 7 days of life.

Grade of MSAF: There are three classifications: grade I (there is light staining, which is translucent in character and light green, or yellow flesh in color), grade II (there is moderate staining, which is opalescent in character and deep green and brown in color), and grade III (there is high meconium staining, which is thick, opaque, and dark green color).

Puerperal Infection: Puerperal infection is a genital tract infection that occurs at any time between the delivery of the fetus and within 6 weeks of the postpartum period. Puerperal infection was considered when the woman had two of the following signs or symptoms: fever (a temperature of 38-39 degree centigrade), chills that accompany fever, lower abdominal pain, uterine tenderness, parametrial tenderness, offensive lochia, and leukocytosis with left shift count [19].

Perinatal Asphyxia: In the study setting, perinatal asphyxia was diagnosed when a newborn's 5th-minute Apgar score was less than 7.

Apgar Score: The Apgar score was calculated using five characteristics: strength and regularity of heart rate, breathing effort, muscle tone and movement, skin color or oxygenation, and reflex response to irritable stimuli. The range of the Apgar score is 0 to 10. A total score between 7 and 10 is regarded as “normal,” whereas a total score below 7 is taken as a “low Apgar score.”

Fetal Heartbeat Monitoring: For high-risk mothers, the fetal heart was monitored using a cardiotocograph (CTG), while the fetal heartbeat of others (mothers without known risk factors) was monitored by intermittent heartbeat auscultation using a fetoscope.

Nonreassuring Fetal Heartbeat Pattern (NRFHRP): NRFHRP was considered when the fetus had at least one of the following conditions: persistent late decelerations, variable decelerations, recurrent prolonged decelerations, persistent bradycardia (fetal heartbeat 100 beats per minute for more than 10 minutes), and persistent tachycardia (fetal heartbeat >180 beats per minute for more than 10 minutes).

Antibiotic Administration during Labor: Antibiotics were given to those mothers who had a prolonged rupture of the membrane (rupture of membrane greater than 12 hours).

Operative Delivery: In this study, operative delivery was considered when the mother delivered either by cesarean section or instrumental delivery (vacuum or forceps delivery).

### 2.7. Data Collection Method and Process

The data was collected by 8 trained midwives and 12 health extension workers working in the catchment area of selected hospitals. The data was collected using interviewer-administered structured questionnaires and a medical chart review, which were adapted from previous studies [13, 20–23].

To check the validity and reliability of the data collection tool, it was pretested on 5% of study participants (laboring mothers with MSAF and CAF) at Woreta and Ebinat primary hospitals prior to the actual data collection. Measurement errors, problem areas, difficult or unclear questions, and response options were modified in the tool after the pretest.

The data was collected in the labor ward, postnatal maternity ward, and NICU (neonatal intensive care unit) in three phases from April to August 2021. Initially, eligible laboring mothers were identified and grouped into two categories. Then, their sociodemographic and some obstetric characteristics were documented. The phone number or mobile number and kebele (lowest administrative unit) of the mothers were registered for later tracing of maternal and perinatal outcomes. In the next phase (phase two), immediate postpartum maternal and neonatal outcomes were documented at the maternity (postnatal) ward and NICU before discharging mothers. Finally, maternal and neonatal outcomes were collected on the 7th postpartum day. Mothers were advised to attend postnatal follow-up by the 7th postpartum day, and maternal and neonatal conditions were checked during this postnatal follow-up. Those mothers who did not have postnatal follow-up by this day were addressed by phone or mobile call. Those mothers who did not have a phone or mobile number were visited by health extension workers who lived in the community to ascertain the status of the mother and their neonate, and then the health extension workers notified the data collectors of the outcomes.

### 2.8. Statistical Analyses

The collected data was examined for missing values and lost to follow-up. Then, the data was analyzed using SPSS version 25. The characteristics of participants were expressed in frequency, percentage, and mean ± standard deviation (SD). Comparisons of categorical variables were performed using chi-squared and Fisher's exact tests. A significant level of difference was considered when a *P* value was lower than 0.05. Relative risk (RR) with 95% confidence intervals was used to determine the strength of the association. Confounders for each maternal and perinatal outcome were managed using multivariable logistic regression. To ascertain their relationship with maternal and neonatal outcomes, all determinant factors of MSAF were chosen for multivariable logistic regression. The statistical significance of the association between covariates and outcomes was determined by using a 95% CI and a *P* value less than 0.05.

## 3. Results

### 3.1. Background Characteristics of Mothers

This study included 580 laboring moms (145 MSAF and 435 CAF cases). There were differences between the two groups by maternal age. The mean maternal age for both the MSAF and clear amniotic fluid groups was 26.56 years, with an SD of ±4.47 years. There were no differences between the two groups by residency, educational status, and occupational status. The majority, 100 (68.96%) of mothers in the MSAF group and 289 (66.43%) of mothers in the CAF group, were from urban areas. Nearly half (53.1%) of the MSAF cases and 251 (57.7%) of the mothers in the CAF groups had primary educational status (Table 1).

### 3.2. Obstetric Characteristics of Study Participants

The mean gestational age (GA) for both the MSAF and CAF groups was 39.61 weeks, with an SD of ±1.168 weeks. There were no differences between the two groups by parity, ANC visit, birth weight and the number of ANC visits, or gestational age. The majority, 141 (97.2%) of the MSAF cases and 422 (97%) cases of CAF, had ANC visits during pregnancy. Regarding parity, 45% of the cases in the MSAF group and 54% of cases in the CAF groups were multiparas. The majority, 99 (68.3%) of mothers in the MSAF group and 91.3% of mothers in the CAF groups, had spontaneous onset of labor. The duration of rupture of the amniotic membrane was less than 12 hours in 138 (95.2%) of the MSAF group and 375 (86.2%) of the CAF group, while seven (4.8% of the MSAF group) and 60 (13.8%) of the CAF group had prolonged rupture of the membrane (>12 hours). Regarding the grade of meconium, 28 (19.32%) were grade I, 57 (39.31%) were grade II, and 60 (41.37%) were grade III (Table 2).

### 3.3. Maternal Outcomes

Wound site infection occurred in 4.1% of the MSAF groups and 1.60% of the CAF groups. However, meconium was not associated with wound site infection. Mode of delivery was significantly associated with MSAF, with operative delivery being more likely to happen in the MSAF group (RR = 5.34, *P* < 0.001). Nearly one-fourth, 41 (28.3%) of mothers in the MSAF group and 23 (5.3%) of mothers in the CAF group, had operative deliveries. Likewise, women with MSAF had a higher risk of developing puerperal sepsis (RR = 5.6,*P* < 0.001). Nearly one-fifth (19.3%) of the MSAF cases had puerperal sepsis, while only 3.4% of the CAF groups had puerperal sepsis (Table 3).

### 3.4. Perinatal Outcomes

There were no differences in the intrapartum stillbirth rate between the two groups (*P* value = 0.1 for the Fisher exact test). Intrapartum stillbirth was found in 2.1% of the MSAF and CAF cases. There was a statistically significant difference in terms of NRFHRP between the CAF cases and the MSAF groups (*P* < 0.001). The risk of NRFHRP was 1.39 times higher in the MSAF cases as compared to CAF. The risk of perinatal asphyxia (PNA) was 1.27 times higher in the MSAF cases as compared to those women with CAF (RR = 1.27, *P* < 0.001). MAS, admission to the NICU, END, and neonatal sepsis were significantly higher in the MSAF group compared with the CAF group. MAS were occurred in 7.58% of the MSAF cases and 0.68% of the CAF groups. Nearly one-fourth (23.4%) of neonates in the MSAF group and 14 (3.2) of neonates in the CAF groups were admitted to the NICU. Regarding neonatal sepsis, 9% of the MSAF cases and 4.1% of the group with CAF had neonatal sepsis (Table 4).

MSAF has a significant association with the onset of labor (induced labor), preeclampsia, IUGR, and oligohydramnios (Table 2) and mode of delivery (operative delivery) in Table 3. So, multivariable logistic regression was done to see the impact of these predictors on maternal and perinatal outcomes. However, none of these factors has a relationship with maternal and perinatal outcomes, and the adverse maternal and perinatal outcomes are only associated with MSAF (Table 5).

## 4. Discussion

This study found that MSAF significantly increased adverse maternal and perinatal outcomes, namely, NRFHRP, perinatal asphyxia, neonatal resuscitation at birth, admission to the NICU, neonatal sepsis, meconium aspiration syndrome, early neonatal death, operative delivery, and puerperal sepsis.

Women with MSAF were more likely to have an operative delivery compared with a clear amniotic fluid group. Studies from St. Paul's Hospital in Ethiopia [17] and the Agha Khan Hospital for Women at Garden Campus [24] found similar evidence and support the current conclusion. In this study, operative delivery was done in 28.3% of the MSAF cases. The present outcome was much less than the evidence obtained from the Agha Khan Hospital for Women-Garden Campus [24], which was 60% and 71% in thin and thick meconium, respectively. This might be because of the different management modalities for MSAF between the study settings. In the previous study, obstetric care providers used aggressive treatment options for MSAF, which increased the rate of CS. However, the management of MSAF was variable across obstetricians in the present study, and most obstetricians considered emergency cesarean section for only grade III MSAF during the latent first stage of labor.

MSAF significantly increased the risk of puerperal sepsis by 5.6 times as compared to clear amniotic fluid. The finding might be justified by the overgrowth of *Escherichia coli* and group B streptococci, which are possible risk factors for puerperal sepsis [25]. Furthermore, the presence of meconium weakens the host's immune system by decreasing the amniotic fluid's antimicrobial characteristics, promoting the growth of microorganisms, and suppressing the inflammatory process. Additionally, if the newborn requires prolonged hospitalization in the NICU due to meconium-related complications, the mother may be at an increased risk of developing postpartum infections due to prolonged hospital stays and exposure to healthcare-associated pathogens. However, this finding is in contrast to previous cohort studies that reported that MSAF had no significant association with puerperal sepsis [21].

This study also found a significant association between MSAF and NRFHRP. Evidence from a previous study supports the present finding and reveals that thick meconium has a significantly greater risk of abnormal FHR tracings [12, 26]. Another study done in Finote Selam primary hospital, Ethiopia, supports the current finding that MSAF was significantly associated with NRFHRP [27]. This might be due to when a baby releases meconium into the amniotic fluid; it can cause changes in the fetal heart rate pattern, leading to a nonreassuring or abnormal pattern.

The incidence of perinatal asphyxia was significantly higher in the MSAF cases when compared to mothers with clear fluid cases. Evidence from Nepal supports the current finding [28]. In the present study, 13.8% of women with MSAF had perinatal asphyxia. Meconium-stained amniotic fluid increases the risk of perinatal asphyxia in different ways: (1) the passage of meconium into the amniotic fluid may interfere with the fetal airway, blocking the airway. This condition might lead to decreased blood flow and/or oxygen to the fetus. (2) Chemical pneumonitis: meconium contains enzymes and other substances that can cause inflammation and damage to the delicate lung tissue. This can lead to chemical pneumonitis, which further impairs the baby's ability to breathe properly. (3) Decreased lung compliance: meconium can also affect surfactant production in the lungs. Surfactant is a substance that helps keep the air sacs in the lungs open and prevents them from collapsing. When meconium interferes with surfactant production, it can result in decreased lung compliance, making it harder for the baby to breathe effectively. (4) Hypoxia and acidosis: aspiration of meconium-stained amniotic fluid can lead to decreased oxygen supply to the baby's body, causing hypoxia (low oxygen levels). This lack of oxygen can result in acidosis, an increased acidity of the blood and tissues. Hypoxia and acidosis can have detrimental effects on various organs, including the brain, heart, and kidneys.

In this study, early neonatal death was more likely to occur in the MSAF group compared to CAF. Evidence at the University Hospital Complex of Vigo [29] and St. Paul's Hospital in Ethiopia [17] supports the present study. The END has occurred in 4.8 percent of the MSAF cases. This finding was higher than a study finding at Duhok Obstetrics and Gynecology Teaching Hospital [30]. This might be due to the differences in early diagnosis and management modalities for high-risk neonates. The presence of meconium in the airways and lungs can cause a blockage, leading to respiratory distress and breathing difficulty. This can result in decreased oxygen levels in the blood, potential damage to vital organs, and finally death.

In the present study, meconium exposure increased the risk of developing MAS. This outcome was consistent with evidence obtained from St. Paul's Hospital [17] and Nepal [28]. In this study, MSA was found in 7.58% of the MSAF cases compared to clear amniotic fluid cases (0.68%). This finding was comparable with a study finding at St. Paul's Hospital [17], with MAS detected in 6.3% of neonates born from MSAF cases. However, this finding was lower than a finding at Duhok Obstetrics and Gynecology Teaching Hospital, which reported 48.1% incidences of MAS in the MSAF group [30]. The reason for this discrepancy could be that the studies used different diagnostic techniques for MAS.

Moreover, there was variation in NICU admissions between the MSAF and CAF groups. This result was in agreement with research from Yenepoya, Mangalore [31], Jawaharlal Nehru Hospital, Bhilai [32], and St. Paul's Hospital in Ethiopia [17]. In this study, 23.4% of neonates in the MSAF group were admitted to the NICU. This result was less than the outcomes obtained from Yenepoya, Mangalore, with 54.7% NICU admission [31] and Jawaharlal Nehru Hospital, Bhilai (50.5% of NICU admission) [32]. The discrepancy might be because of the sampling size variation between studies. In the previous study, a total of 350 and 200 MSAF cases were involved at Yenepoya, Mangalore [31], and Jawaharlal Nehru Hospital, Bhilai [32], respectively. The increased risk of admission to the NICU in cases of meconium-stained amniotic fluid might be due to meconium aspiration syndrome, infection risk, respiratory complications, low oxygen levels, difficulty maintaining body temperature, feeding difficulties, and other medical concerns.

Lastly, neonatal sepsis was more likely to occur in the MSAF cases compared to clear amniotic fluid cases. Similar outcomes were reported in Cameron by Dohbit et al. [21]. The high incidence of neonatal sepsis in the MSAF case might be due to meconium, which decreases the antimicrobial effects of amniotic fluid and facilitates the overgrowth of microorganisms that cause neonatal sepsis [25]. Meconium-stained amniotic fluid increases the risk and development of neonatal sepsis in the following ways: (1) meconium can harbor bacteria and other microorganisms, which can enter the infant's bloodstream during delivery or through aspiration into the lungs. These microorganisms can cause sepsis if they are not adequately cleared from the infant's system. (2) Meconium aspiration syndrome (MAS) occurs when a newborn inhales meconium-stained amniotic fluid into the lungs. This can lead to respiratory distress and compromise the infant's ability to fight off infections. (3) Meconium contains various substances that can impair the immune response of the newborn. (4) Meconium can trigger an inflammatory response in the newborn's body. This inflammation can contribute to the development and progression of sepsis.

This study has some limitations. Certain maternal and neonatal outcomes might go unnoticed due to the short postpartum follow-up period (7 days). Besides this, some study participants were traced by mobile phone after being discharged from the hospital. This might lead to inaccurate information about maternal and neonatal outcomes.

## 5. Conclusions

Meconium-stained amniotic fluid significantly increased adverse maternal and perinatal outcomes in Ethiopia. The risk of perinatal asphyxia, nonreassuring fetal heart rate pattern, neonatal sepsis, meconium aspiration syndrome, admission to the NICU, early neonatal death, operative delivery, and puerperal sepsis were significantly higher in meconium-exposed groups. We recommend strict intrapartum and postpartum monitoring of MSAF cases with the aim of timely detection and treatment of the negative effect of meconium. In addition, special attention should be given to meconium-exposed mothers in the postnatal follow-up, and mothers should be advised on early postnatal follow-up. Future studies may examine the long-term implications of MSAF. Lastly, to categorize the treatments appropriately, we advise well-controlled research comparing maternal and neonatal outcomes for each grade of MSAF.

## Figures and Tables

**Table 1:** Background characteristics of laboring mothers.

Characteristics	MSAF (*n* = 145) *N* (%)	CAF (*n* = 435) *N* (%)	Chi-square (*P* value)
Age (in year)			11.881 (0.008)
≤20	20 (13.8%)	39 (9%)	
21-25	64 (44.1%)	241 (55.4%)	
26-30	38 (26.2%)	70 (16.1%)	
>30	23 (15.9%)	85 (19.5%)	
Residency			
Urban	100 (69%)	289 (66.4%)	1.112 (0.575)
Rural	45 (31%)	146 (33.6%)	
Educational status			1.104 (0.766)
Nonformal education	4 (2.8%)	12 (2.8%)	
Primary	77 (53.1%)	251 (57.7%)	
Secondary	37 (25.5%)	95 (21.8%)	
College and above	27 (18.6%)	77 (17.7%)	
Occupation			2.880 (0.41)
Housewife	84 (57.9%)	263 (60.5%)	
Merchant	41 (28.3%)	96 (22.1%)	
Civil servant	11 (7.6%)	45 (10.3%)	
Farmer	9 (6.2%)	31 (7.1%)	

**Table 2:** Obstetric characteristics for women with MSAF compared with CAF.

Characteristics	MSAF (*n* = 145) *N* (%)	CAF (*n* = 435) *N* (%)	Chi-square (*P* value)
Parity			3.152 (0.27)
Primipara (1)	51 (35.2%)	129 (29.7%)	
Multipara (2-4)	66 (45.5%)	235 (54%)	
Grand multipara (≥5)	28 (19.3%)	71 (16.3%)	
ANC follow-up	141 (97.2%)	422 (97%)	0.20 (1.00^∗^)
Number of ANC visits (*n* = 563)			4.479 (0.107)
≤2	20 (13.8%)	35 (8%)	
>2	120 (82.8%)	388 (89.2%)	
GA in weeks			4.973 (0.083)
37-38	19 (13.1%)	88 (20.2%)	
39-40	104 (71.7%)	270 (62.1%)	
>40	22 (15.2%)	77 (17.7%)	
Fetal weight (kg)			0.88 (0.644^∗^)
<2.5 kg	8 (5.5%)	24 (5.5%)	
2.5-4 kg	135 (93.1%)	399 (91.7%)	
>4 kg	2 (1.4%)	12 (2.8%)	
Onset of labor			
Spontaneous	99 (68.3%)	397 (91.3%)	
Induced	46 (31.7%)	38 (8.7%)	46.40 (<0.001)
Preeclampsia	23 (15.9%)	31 (7.1%)	9.82 (0.002)
IUGR	17 (11.7%)	5 (1.1%)	33.32 (<0.001)
APH	7 (4.8%)	25 (5.7%)	0.176 (0.674)
Oligohydramnios	11 (7.6%)	5 (1.1%)	16.79 (<0.001^∗^)
GDM	6 (4.1%)	9 (2.1%)	1.84 (0.174^∗^)
Chorioamnionitis	2 (1.4%)	13 (3%)	1.11 (0.29^∗^)

^∗^Fisher's exact *P* value for small size values.

**Table 3:** Maternal outcomes of laboring mothers with CAF and MSAF.

Characteristics	MSAF (*n* = 145) *n* (%)	CAF (*n* = 435) *n* (%)	RR with 95% CI	*P* value
Mode of delivery				
Spontaneous vaginal	104 (71.7%)	412 (94.7%)	0.75 (0.68, 0.84)	
Operative delivery	41 (28.3%)	23 (5.3%)	5.34 (3.32, 8.59)	<0.001
Wound site infection	6 (4.1%)	7 (1.6%)	2.57 (0.87, 7.52)	0.10^∗^
Puerperal sepsis	28 (19.3%)	15 (3.4%)	5.6 (3.07, 10.18)	<0.001

^∗^Fisher's exact *P* value for small size values.

**Table 4:** Fetal and neonatal outcomes of laboring mothers with MSAF and CAF.

Characteristics	MSAF (*n* = 145) *n* (%)	CAF (*n* = 435) *n* (%)	RR with 95% CI	*P* value
Intrapartum stillbirth	3 (2.1%)	9 (2.1%)	1 (0.27, 3.64)	1.0^∗^
Nonreassuring fetal heart rate pattern	43 (29.7%)	9 (2.1%)	1.39 (1.25, 1.54)	<0.001
5th minute Apgar score <7	33 (22.8%)	8 (1.8%)	1.27 (1.16, 1.39)	<0.001
Perinatal asphyxia	33 (22.8%)	8 (1.8%)	1.27 (1.16, 1.39)	<0.001
MAS	11 (7.58%)	3 (0.68%)	11 (3.11, 38.8)	<0.001^∗^
END	7 (4.8%)	6 (1.4%)	3.5 (1.19, 10.24)	0.015^∗^
Neonatal sepsis	13 (9%)	18 (4.1%)	2.16 (1.08, 4.31)	0.025
Admission to NICU	34 (23.4%)	14 (3.2%)	7.2 (4.02, 13.18)	<0.001

^∗^Fisher's exact *P* value for small size values.

**Table 5:** Multivariable logistic regression of factors associating with MSAF and maternal and perinatal outcomes.

Maternal and perinatal outcomes	Independent variable	AOR (95% CI)	*P* value
Operative delivery	Induced labor	1.08 (0.51, 2.29)	0.822
	Preeclampsia	1.46 (0.65, 3.26)	0.35
	IUGR	2.48 (0.88, 6.98)	0.084
	Oligohydramnios	2.23 (0.65,7.67)	0.2

Puerperal sepsis	Induced labor	1.67 (0.75, 3.71)	0.20
	Preeclampsia	2.25 (0.97, 5.21)	0.58
	IUGR	2.28 (0.99, 5.72)	0.056
	Oligohydramnios	1.34 (0.32, 5.59)	0.687
	Operative delivery	2.07 (0.92, 4.64)	0.076

Perinatal asphyxia	Induced labor	1.57 (0.76, 3.25)	0.221
	Preeclampsia	2.09 (0.95, 4.59)	0.064
	IUGR	0.65 (0.13, 3.15)	0.595
	Operative delivery	1.95 (0.92, 4.12)	0.08
	Oligohydramnios	0.42 (0.05, 3.38)	0.421

NRFHRP	Preeclampsia	2.02 (0.98,4.43)	0.072
	IUGR	1.20 (0.33, 4.27)	0.777
	Induced labor	1.79 (0.88,3.64)	0.105
	Oligohydramnios	1.54 (0.40, 5.93)	0.527

MAS	Induced labor	3.13 (0.95, 10.30)	0.06
	Preeclampsia	2.69 (0.95,6.23)	0.99
	IUGR	1.48 (0.15, 14.12)	0.73
	Operative delivery	2.31 (0.62, 8.65)	0.211
	Oligohydramnios	1.86 (0.19, 17.57)	0.58

END	Induced labor	2.70 (0.81, 8.99)	0.104
	Preeclampsia	2.26 (0.54, 9.47)	0.262
	Operative delivery	2.35 (0.62, 8.86)	0.205
	IUGR	1.35 (0.14, 12.61)	0.792

Neonatal sepsis	Induced labor	0.48 (0.11, 2.10)	0.331
	Preeclampsia	0.29 (0.03, 2.20)	2.33
	IUGR	1.45 (0.17, 12.38)	0.733
	Operative delivery	2.03 (0.80, 5.15)	0.136

Admission to NICU	Induced labor	1.47 (0.68, 3.18)	0.317
	Preeclampsia	1.20 (0.46, 3.10)	0.698
	IUGR	1.20 (0.30, 4.81)	0.794
	Oligohydramnios	2.66 (0.73, 9.68)	0.137
	Operative delivery	1.09 (0.44, 2.69)	0.852

## Data Availability

The corresponding author will provide the dataset and any additional sources utilized in this research upon reasonable request.

## Abbreviations

ANC: Antenatal care
APH: Antepartum hemorrhage
CAF: Clear amniotic fluid
CS: Cesarean section
DTU: Debre Tabor University
GA: Gestation age
GDM: Gestational diabetes mellitus
IUGR: Intrauterine growth restriction
MAS: Meconium aspiration syndrome
MSAF: Meconium-stained amniotic fluid
NICU: Neonatal intensive care unit
END: Early neonatal death
NRFHRP: Nonreassuring fetal heart rate pattern
PNA: Perinatal asphyxia
RR: Relative risk.
